# THE GOOD, THE BAD, AND THE BALANCE: CHALLENGES AND OPPORTUNITIES IN USING TECHNOLOGY IN DEMENTIA CARE DYAD

**DOI:** 10.1093/geroni/igae098.1410

**Published:** 2024-12-31

**Authors:** Kuan-Hua Chen, Lyndsey Miller, Allison Lindauer

**Affiliations:** Chair; Co-Chair; Discussant

## Abstract

Advances in modern technologies, including wearables, mobile or embedded devices and sensors, and AI provide researchers and clinicians with exciting opportunities for naturalistic, large-scale, long-term, and timely assessment and intervention. In dementia, the vast majority of research and interventions using technology have been focusing on individuals—either only the care recipient or their family care partners. However, most dementia care dyads live together and have interdependent behaviors (e.g. physical activity and sleep) and shared functions (e.g. preparing meals). The proposed symposium highlights recent advances in using technologies to assess or intervene in dementia care dyads including their dyadic interactions and interdependent behaviors and functions in their shared home space. The five presentations will leverage different technologies (i.e., motion and door sensors, bed mat sensors, smartwatch, AI) to assess or intervene in different aspects of the health of dementia care dyad (e.g., loneliness, exercise, sleep, worrisome behaviors). Being aware that any methodology also has limitations and pitfalls, each presentation will include an instructive review of challenges associated with the technologies and efforts to overcome them. The five individual presentations will be followed by an integrated panel discussion, moderated by Dr. Harleah Buck. Together, the symposium will provide the general audience with an overview of current in-home assessment technologies, the challenges to incorporating them, and the tradeoffs for science. This symposium will also launch communication and collaborations between researchers from different disciplines who use modern technologies to conduct research in naturalistic settings to promote the health and well-being of dementia care dyads. Dyadic Health Research Interest Group Sponsored Symposium

